# Discrete element modeling of particles sphericity effect on sand direct shear performance

**DOI:** 10.1038/s41598-022-09543-9

**Published:** 2022-03-31

**Authors:** Chunhui Chen, Jiayu Gu, Zesen Peng, Xianyao Dai, Qingbing Liu, Guo-Qiang Zhu

**Affiliations:** 1 Badong National Observation and Research Station of Geohazards (BNORSG), Three Gorges Research Center for Geo-Hazards of Ministry of Education, China University of Geosciences, Wuhan, 430074 China; 2 Faculty of Engineering, China University of Geosciences, Wuhan, 430074 China; 3 Hubei Provincial Communications Planning and Design Institute CO., LTD., Wuhan, 430051 China

**Keywords:** Civil engineering, Solid Earth sciences

## Abstract

Particle surface morphology is an important factor influencing sand structure and mechanical properties. In this study, the effect of sand particle sphericity on sand direct shear performance is investigated by using the discrete element method (DEM). Two ways are adapted to simulate different approaching methods from round particles to irregular sand. The macroresponse shows that irregular sand has a higher shear strength at lower normal stress than round particles. The shape of the particle has less influence on shear strength at higher normal stress. The irregular shape of sand leads to an increase in the shear band proportion. However, the shear band proportion is not related to the sphericity. Under all conditions, particles within the shear band have a larger average rotation angle than those outside the shear band. When the particle shape approaches round (regardless of the round particle proportion and particle shape), the average rotation angle of particles within and without shear bands increase, while the coordinate number and contact anisotropy decrease.

## Introduction

Coarse-grained soil is an aggregate of individual particles that range from round to angular. It consists of a large number of different shapes, sizes, and particle arrangements. Particle surface morphology is an important factor influencing sand structure and mechanical properties^1–4^. Conventional geotechnical tests can only reflect the stress–strain relationship of coarse-grained soils at the macroscopic level. However, the microscopic performance, including particle rotation, displacement, relocation and contact behavior during the tests, is still unclear^5^. The discrete element method (DEM) is an effective method to simulate granular material performance. In the discrete element method, particles are normally simplified to be discs (2D) or spheres (3D), which can reduce computing time and improve working efficiency. However, particles show excessive rotation due to the smooth surface morphology, and the real particle contacts are much different from the spherical ball contacts. The irregularity of the particle shape influences the soil compressibility, the internal friction angle and the interparticle contact force^6,7^. To model different particle shapes, typical particle shapes are generated by discs^8^, ellipses^9^ and polygons^10,11^ in two dimensions, and spheres^12^, polyhedrons^13^ in three dimensions. These particular types of particles can show different shear performances between round balls and irregularly shaped sand^14,15^. To quantitatively analyze particle surface morphology, some measurable indices including particle roundness^16^, sphericity^17^, roughness^18^, angularity^19^ and convexity^20^ are applied to particle geometry. Sphericity can quantify the degree of similarity between a particle and a ball. It is closely related to particle rotation and rearrangement which are crucial to granular material macrobehavior^21^. The cumulation of particle rotation and rearrangement lead to the shear band development and localized failure^22^. The effect of particle shape on granular materials has been explored in recent years, but little work has been carried out to understand particle behavior in the shear band region at the microscale level.

This study focuses on the effect of particle sphericity on sand shear properties, especially in the shear band region. Sand particle shapes are first captured by microscopy, drawn by CAD, and modeled by the particle flow code (PFC^2D^) with irregular shapes and an ideal roundness disc. Experimental tests are conducted in parallel to validate the simulation results. The micro behavior analysis of the particle shape effect on shear performance, including the shear stress–strain curve, shear band behavior, particle rotation and contact, is analyzed in detail in the following section.

## Methodology

### Sand morphology capture and modeling

**Figure 1:**
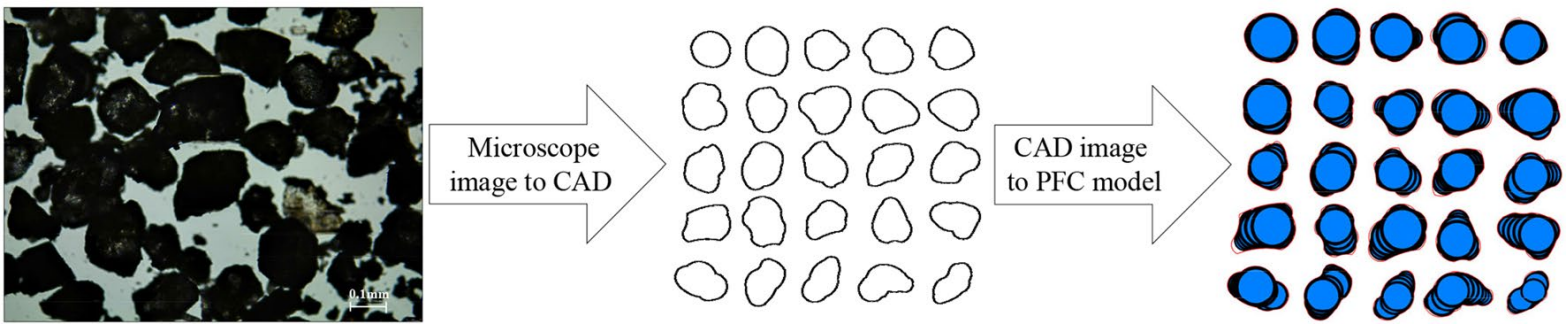
Schematic diagram of sand particles PFC modeling process.

**Figure 2:**
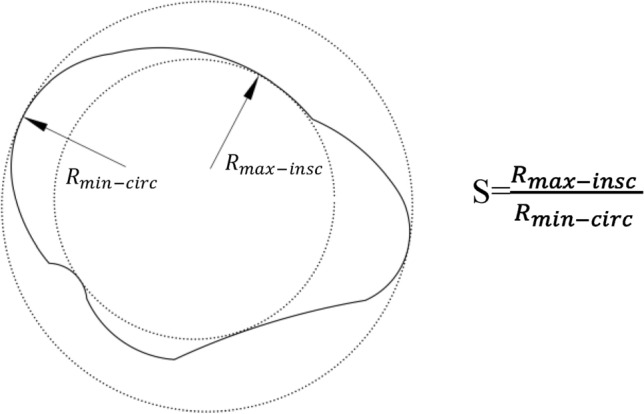
Description of sphericity.

In PFC, realistic particles can be modeled by different assembled clumps with different shapes. Sand particles have unique shape characteristics, but 25 randomly selected sand shapes can generally represent overall particle shape characteristics in geotechnical tests^23,24^. Thus, in this study, 25 randomly selected sands were chosen and observed using a microscope. Their particle shapes were captured and displayed. Then, particle images were imported into CAD software to draw outlines. Clump templates can be formed by the pebble in these outlines through the PFC built-in function. Thus, different shapes of particles were generated by irregular clumps. The corresponding schematic diagram is presented in Fig. 1. Then, these 25 irregular clump templates were generated in PFC^2D^ according to their particle size distribution, which can be regarded as natural sand shapes. Sphericity quantifies the degree of similarity between a particle and a sphere^1,25^. In 3D, the ture sphericity was the ratio of surface area of a sphere of the same volume as the particle to the actual exterior surface of the particle^26^. However, the measurement of three-dimensional (3D) grain surface is quite challenging and practically impossible. Thus, Wadell^27^ proposed diameter sphericity which convererted the 3D particle sphericity into 2D plane projection of the particle. Rorato et al.^28^ compared particle diameter sphericity and ture sphericity by using Hostun and Caicos sand, a good correlation can be found between these values. Thus, the diameter sphericity is adapted in this research and calculation method is presented in Fig. 2. The results show sand particle sphericity value (S) ranges from 0.52 to 0.87. Ideal round sand with a corresponding sphericity value of 1 is generated by a disc; this is set as the control group.

**Figure 3:**
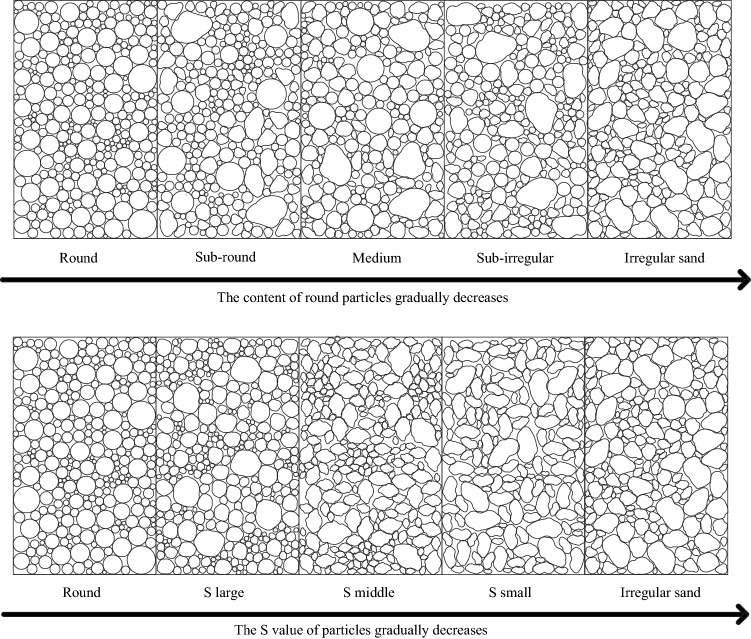
Schematic of sand particle modeling with different shapes.

Then, two simulation methods were adapted to explore the particle sphericity effect from round discs to irregular sand. One method involved mixing round discs with irregular clump in different proportions (100% round disc, 75% round disc 25% irregular clump, 50% round disc 50% irregular clump, 25% round disc 75% irregular clump, 100% irregular clump) to explore the effect of particle shape with different proportions. They are named round (100% round disc), subround (75% round disc 25% irregular clump), medium (50% round disc 50% irregular clump), subirregular (25% round disc 75% irregular clump), and irregular (100% irregular clump). The other method used one assigned type of clump with a typical S value as the replacement for a round disc to explore the single particle shape effect. They are named S large (S = 0.87), S middle (S = 0.69), and S small (S = 0.52). Details of the schematic diagram can be seen in Fig. 3.

### Direct shear test modeling and parameters calibration

**Figure 4:**
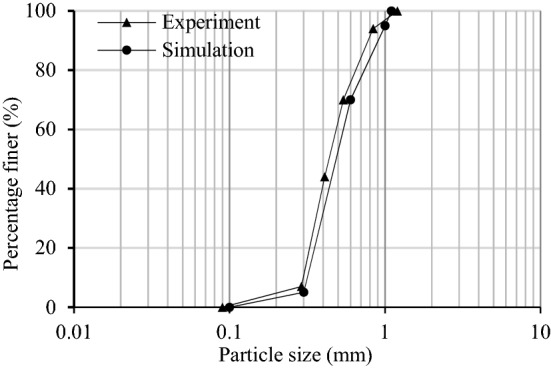
Particle size distribution.

**Figure 5:**
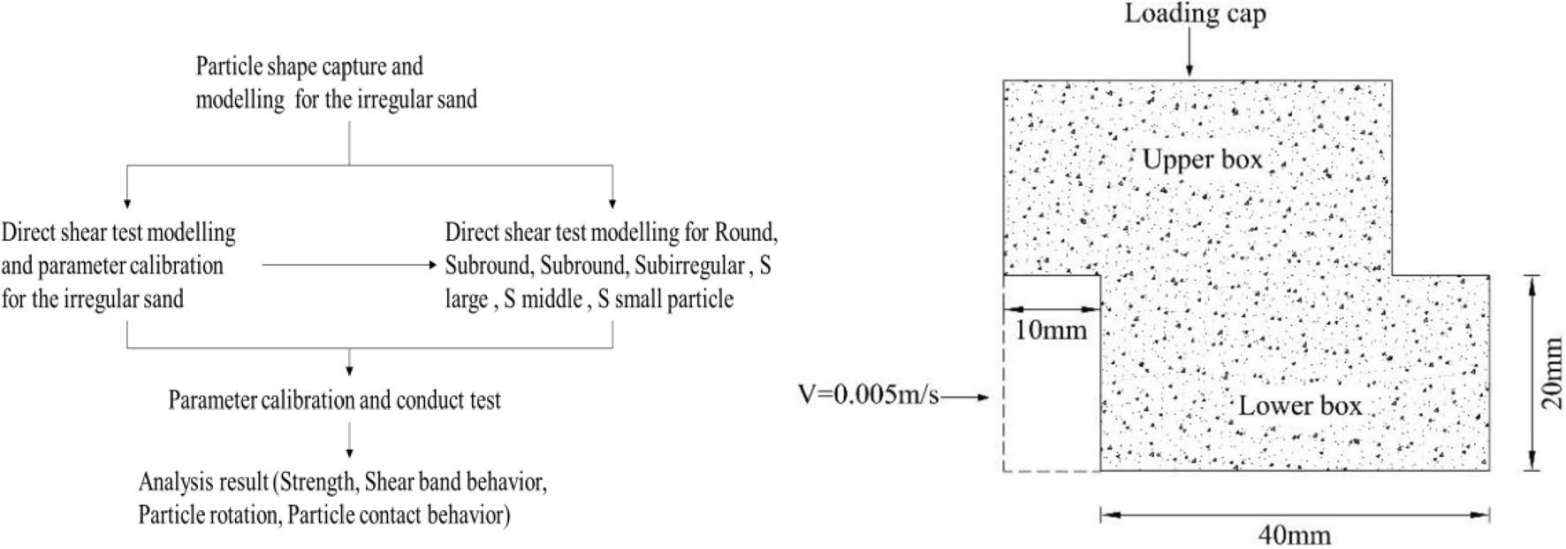
The schematic diagram of conducted test.

To benchmark the results from the corresponding numerical simulation, direct shear tests are carried out in parallel to calibrate the PFC input parameters. Clean and uniform sand with a specific gravity of 2.65 was chosen as the basic material in this research. The particle size distribution of the soil for the experimental and numerical tests is listed in Fig. 4. As the shear box width should be more than 10 times the maximum particle size and the shear box height should be more than 6 times the maximum particle size^29^, the shear box’s dimensions for both the experimental direct shear test and numerical shear test was 40 mm * 40 mm. Sand was poured into the shear box with a dry density of 1.59 g/cm^3^. Loading speeding was set as 1% strain per minute. Direct shear tests were performed under normal stresses of 50 kPa, 100 kPa, and 200 kPa. All tests were conducted under the guidance of the ASTM D3080-04 and Chinese standard GB/T 50123-2013. For the numerical direct shear test, the shear box is modeled by the enclosed wall. The top wall is treated as a servo wall with a vertical velocity until the normal stress reaches the target value of 50 kPa, 100 kPa, or 200 kPa. When shear velocity is conducted at different velocities in the PFC, shear result differences are indistinct when the shear velocity is less than 0.1 m/s. This velocity is much different from the experimental test, as particle contacts may dissipate kinetic energy due to the damping acting^8^. Thus, the lower part of the shear box is made up of three walls, which are given a constant speed of 0.005 m/s, while the upper part of the shear box remains stationary. The stress on the vertical walls is recorded as shear stress, which is similar to the experimental test. It should be noted during the numerical simulation of the shear test that a horizontal plate is added to avoid particle leakage. The schematic diagram of conducted test is illustrated in Fig. 5.

**Figure 6:**
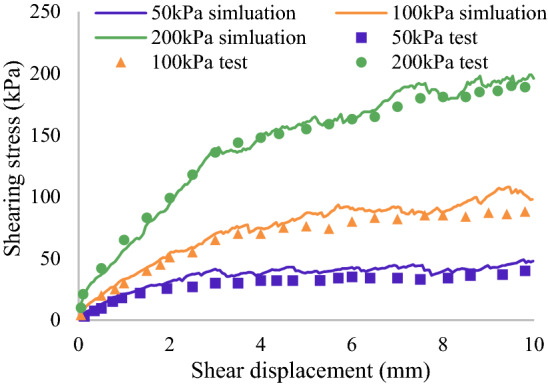
Shear stress–strain curve of experimental test and numerical simulation for clean sand.

The contact model between the cohesionless sand is applied by the rolling resistance linear model^30^. The rolling resistance model is a linear-based model that adds a rolling resistance mechanism. The model incorporates a torque acting on particle contact to counteract rolling motion and to dissipate energy during the relative rotation^31,32^. The rolling resistance model can offer a simple method to simulate particle shape-like behavior^33^. Following the proposed method by Lu et al.^8^, Young’s modulus and friction coefficient are roughly calibrated. Then, a numerical direct shear test is conducted to match the experimental data. Figure 6 presents the shear stress–strain curve of the experimental test and numerical simulation for clean sand samples. The input parameters of the shear test are listed in Table 1.

**Table 1:** Input parameters of shear test.

Parameters	Value
Soil density (kg/m^3^)	2650
Soil contact Young’s modulus (Pa)	1 * 10^7^
Soil particle static friction coefficient	0.4
Soil particle rolling friction coefficient	0.05
The ratio of normal to shear stiffness K_n_/K_s_	1
Damping coefficient	0.5
Normal critical damping ratio	0.1
Shear critical damping ratio	0.1
Shear velocity (m/s)	0.005

## Results and discussion

### Shear strength of different particle assembles

**Figure 7:**
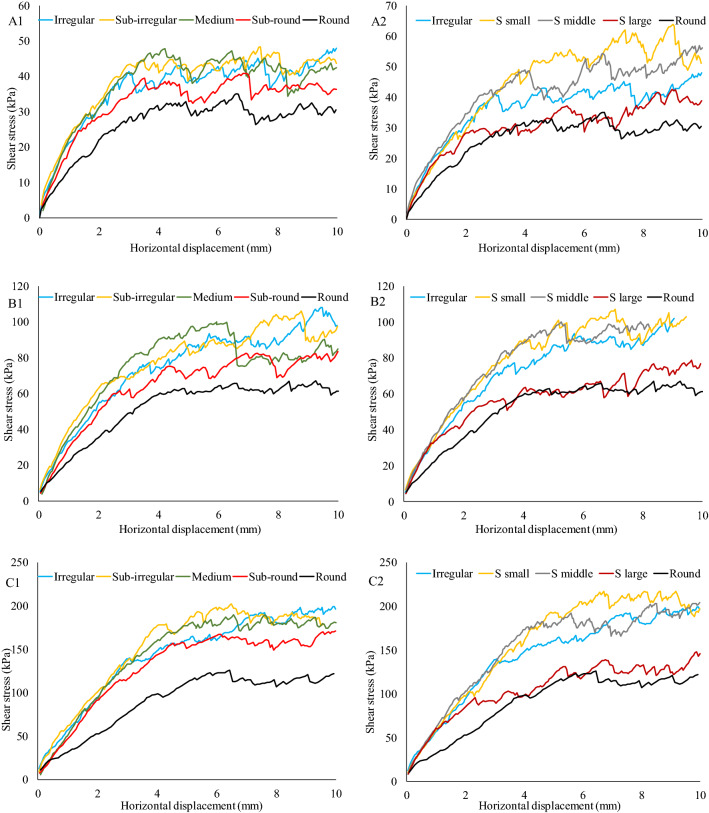
Stress–strain curve for all cases (A1-C1 are the mixture of round and irregular clump under 50 kPa, 100 kPa and 200 kPa normal stress; A2-C2 are the assigned types of clump with different S value under 50 kPa, 100 kPa and 200 kPa normal stress).

The shear strength of sandy soils is mainly influenced of friction and interlocking structures between particle surfaces. Even under the same test conditions, the shear stress-displacement curves also present different trends. Figure 7 presents the shear stress-displacement curve for all cases under 50 kPa, 100 kPa and 200 kPa normal stress. At a lower normal stress (50 kPa), round particles show the lowest shear stress when compared with mixed soil or with a typical shape clump. There is a clear increasing trend of shear stress for the mixed soil. However, this increasing trend is unrelated to the round particle proportion. The medium (50% round disc 50% irregular clump), subirregular (25% round disc 75% irregular clump), and irregular (100% irregular clump) soil present similar shear stress curves, while subround (75% round disc 25% irregular clump) shows smaller stress. For the single assigned type of clump, the shear stress increases as the S value decreases^34^. Even though particles have similar grain sizes and gradations, irregular particle shapes lead to a higher shear strength. At higher normal stresses (100 kPa and 200 kPa), the shear strength of mixed soil is still much higher than that of round particles. Even a small proportion (75% round disc 25% irregular clump) of irregular soil can significantly increase the soil shear strength. However, for the single assigned type of clump, particles with a larger S value exhibit little improvement in shear strength at higher normal stress. This indicates that when the particle approaches a round shape, its shape has less influence on shear strength at higher normal stress.

### Shear band behavior

**Figure 8:**
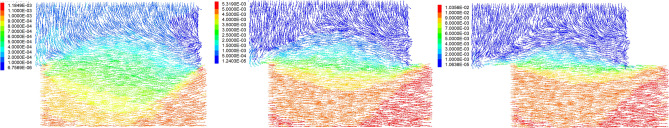
Irregular clump displacement vector at 50 kPa at 1 mm, 5 mm and 10 mm shear displacement.

When shear advances, the movement of coarse-grained particles results in particle relocation. The stress redistribution is adjusted by changing the shear displacement. The macromechanical shear performance of sand is the result of the microstructure’s evolution during shear^35^. Figure 8 presents the irregular clump particle displacement vector at 50 kPa with shear displacements of 1 mm, 5 mm, and 10 mm. Different colors represent different displacement values, while the vector indicates the particle movement path. Particles in the lower shear box show larger horizontal displacement because they move with the lower shear box. Particles in the upper shear box exhibit two directions of movement: particles are forced to move downward at the top, while particles near the shear plane move horizontally. Particles near the shear plane show different displacement behaviors, but all present a bell-shaped curve. Soil particles with large displacement near the shear plane can be named shear bands or shear zones^36^. The localization of shear deformations and shear band formation contribute to the destabilization of soil. However, the traditional direct shear test mainly focuses on the macroscopic mechanical response. It is almost impossible to directly observe the behavior of particles in the shear zone.

**Figure 9:**
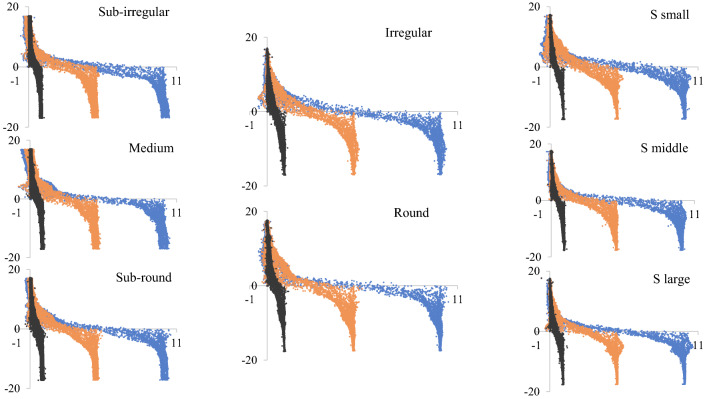
Soil particle horizontal displacement distribution for all cases under 50 kPa normal stress at shear displacement of 1 mm, 5 mm and 10 mm.

Thus, we quantitatively calculated the horizontal displacement of all particles in the shear box. Figure 9 presents the distribution of the soil particles’ horizontal displacement for all cases under 50 kPa normal stress with shear displacements of 1 mm, 5 mm and 10 mm. The horizontal axis shows the particle’s horizontal displacement, while the vertical axis shows the particle’s vertical position in the shear box. The closer the particles are to the shear plane, the larger the horizontal displacement. Particles in the lower shear box mobilize with the shear box simultaneously; thus, the displacement of most particles in the lower shear box is similar to their horizontal movement.

To visualize shear band shape, horizontal displacement (*L*_*i*_) of the sand was recorded. If *Li/Ls* ≥ 0.1 (*Ls* is shear displacement at the moment), this soil particle can be regarded as being within the shear band. Details are listed as follows.

$$\left\{\begin{array}{ll}\left|\frac{{L}_{i}}{{L}_{s}}\right|\ge 0.1, & \quad when \; soil \; particles \; locat \; in \; the \; upper \;shear \;box \\ \left|\frac{{L}_{i}-{L}_{s}}{{L}_{s}}\right|\ge 0.1, & \quad when \;soil \;particles \;locat \; in \; the \; lower \; shear \;box\end{array}\right.$$

**Figure 10:**
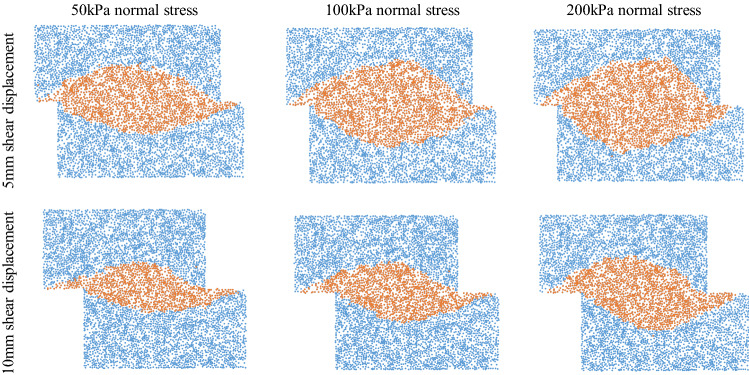
Reconstruction images of shear band distribution of irregular sand.

Then, the position information of the shear band particle was used to reconstruct the shape of the shear band in the scatter plot. Figure 10 presents the reconstruction images of the shear band distribution for irregular sand.

**Figure 11:**
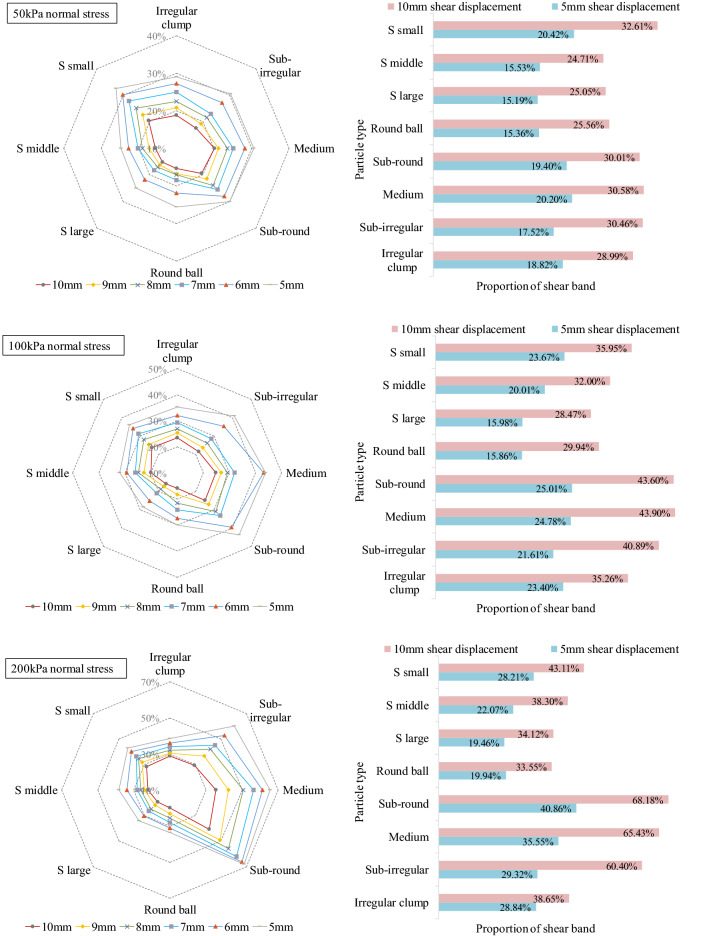
Shear band proportion of all shape particles under the shear.

The shear band is usually simplified to have a rectangular shape. Formation of a shear band is universal. However, the shear band in the shear box has a fusiform shape with a large area in the middle. Zhou et al.^37^ linked shear band width with rotation. Samples expanded with the development and reached stability. Vangla and Latha^38^ estimated the shear band thickness from the initial and final profiles of colored sand columns. Although the shear band can be visualized, a quantitative definition of the shear band range is still lacking. To quantitatively analyze the shear band performance, the shear band proportion defined as the ratio of shear band particles to the whole particles in the shear box. The calculated shear band proportion is listed in Fig. 11. The left figures present the shear band proportion of all cases from 5 to 10 mm shear displacement under 50 kPa, 100 kPa and 200 kPa. The dashed line and corresponding percentage show the shear band proportion, while different colored solid lines show each particle’s assembled shear displacement. The right figures list details of the shear band proportion that have shear displacements of 5 mm and 10 mm. The shear band proportion is not a constant value for each specimen; it changes according to the shear displacement. The irregular shape of sand leads to an increase in the shear band proportion. When shear advances, the rotation and movement of particles gradually become stable. It can be likely concluded that the shear band area increases with increasing shear displacement at each normal stress. As the normal stress increases, more particles are compacted into a dense state, which hinders particle movement during shear. Thus, the shear band area also increases. The irregular particles have a larger shear band area than the round particles. However, the shear band proportion is not related to the sphericity. There is no clear change trend of the shear band proportion from round to irregular particles. It should be noted that a mixture containing irregular and round sand increases the shear proportion significantly, especially at higher normal stresses.

### Particle rotation

**Figure 12:**
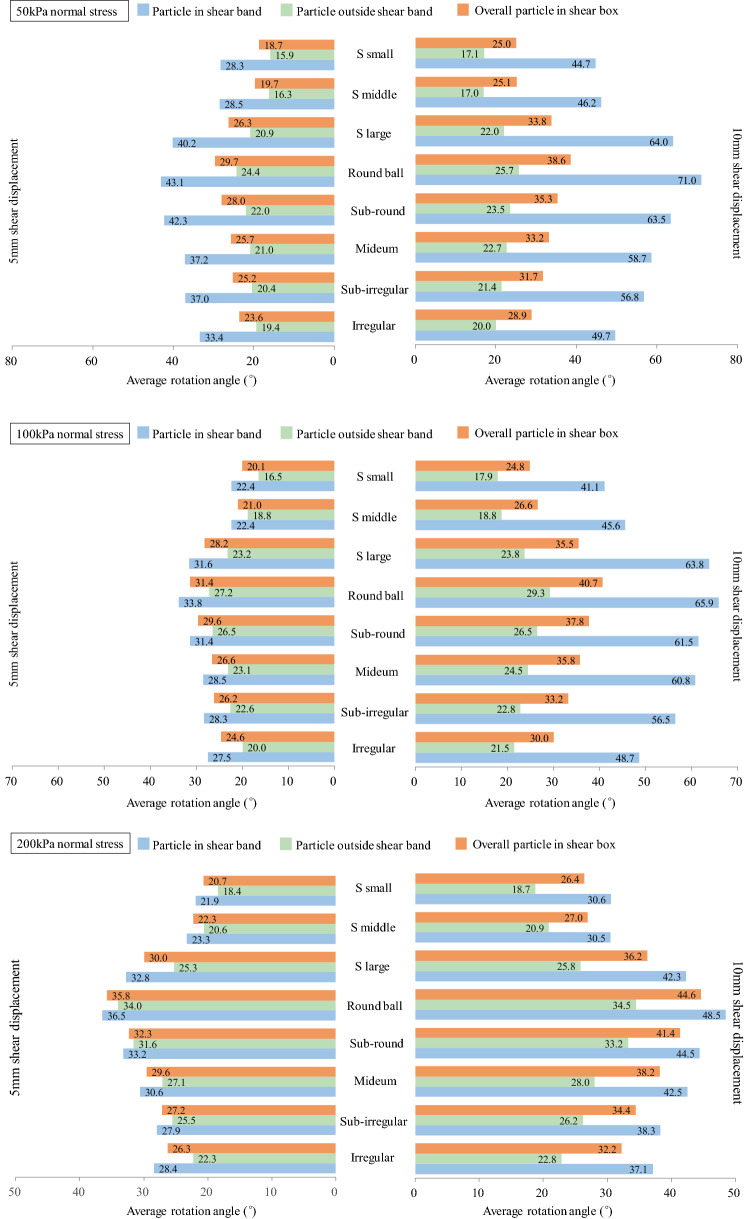
Particle average rotation angle in 5 mm and 10 mm shear displacement.

Particle resistance to rotation is known to develop in particle contact and contribute to enhancing the shear strength. As the shear band position and outline have been quantitatively obtained in the previous section, we classify particles into three groups: particles in the shear band, particles outside the shear band, and overall particles in the shear box. The corresponding average rotation angles are obtained and calculated. Figure 12 presents the average particle rotation angle at shear displacements of 5 mm and 10 mm under 50 kPa, 100 kPa and 200 kPa normal stresses. In all conditions, regardless of shear displacement and normal stress, particles within the shear band have a larger average rotation angle than those outside the shear band. When the particle shape begins to round (regardless of the round particle proportion and particle shape), the average rotation angle of particles with and without shear bands increases. This indicates that an irregular particle shape reduces particle rotation. In addition, compared with outside shear band particles, the average particle rotation angle within the shear band presents larger differences for each case, indicating that the particle shape has a larger influence on shear band particles. When shear advances from a 5 mm to 10 mm shear displacement, more particles are disturbed. Thus, a larger rotation angle is obtained. For round particles, the average contact number is small, as only one contact can be generated between two individual particles. The interparticle structure has difficulty resisting the moment caused by the tangential contact force, leading to a higher particle rotation. For irregular soil, the average contact number between particles increases, and the interparticle structure is more stable. Thus, the moment caused by the tangential contact force can be transmitted between the particles without causing excessive particle rotation. The excessive rotation of particles leads to a lower shear strength, which is confirmed in Fig. 7. The interlocking structure of irregular sand surfaces restrains and reduces particle rotation, resulting in a higher load resistance. When considering the effect of normal stress on particle rotation, it can be concluded that a higher normal stress increases the overall particle rotation angle. However, in the shear band, particles rotate less at a higher normal stress. When the normal stress increases, particle movement needs to overcome more external resistance, which leads to the reduction of particle rotation in the shear band.

### Particle contact behavior

**Figure 13:**
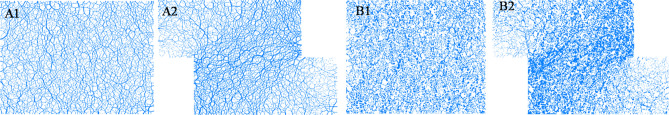
Contact force chain distribution before and after shear (A: round particle; B: irregular clump).

In the PFC simulation, particles interact with others at contacts by generating internal forces. Individual particles are treated as rigid bodies in PFC. Thus, all deformations occur at the contacts. Though granular materials can be regarded as interacting particles, the particle contact force and orientation can determine the microstructure of the soil^39^. For the sand particle, forces are conveyed to one another by their contacts. Figure 13 presents the contact force chain distribution for round particles and irregular clumps before and after the test under 50 kPa normal stress. Force chain thicknesses are proportional to their magnitude. Before shearing, samples are subjected to compression force, as all samples are contained by the shear box. Because the shear box is subjected to vertical force, the compression force in the perpendicular direction is higher than that in the horizontal direction. When the lower box starts to move, sand particles near the moving wall are forced to move. The contact force redistributes to be more diagonally oriented from the lower left-hand corner to the upper right-hand corner. In addition, the contact force increases with higher angularity^40^. When particles approach irregularity, they are more discrete because more contacts can be formed between the irregular particles. Thus, the increase in contact force can be formed in irregular sand with a high interlocking structure, which is in line with Yang et al.^41^.

For the particles, the macrobehavior of shear strength mainly depends on the initial density and the normal stress, while the average contact number is another factor from a micro perspective^42^. The coordinate number is the number of particles in contact with the surrounding solids, and the average coordination number is the basic microscopic index of the granular material. The average coordination number (*Z*) is defined as

1 $$ Z = {2}N_{c} /N_{p} $$

where *N*_*c*_ is particle contact number, *N*_*p*_ is particles number.

**Figure 14:**
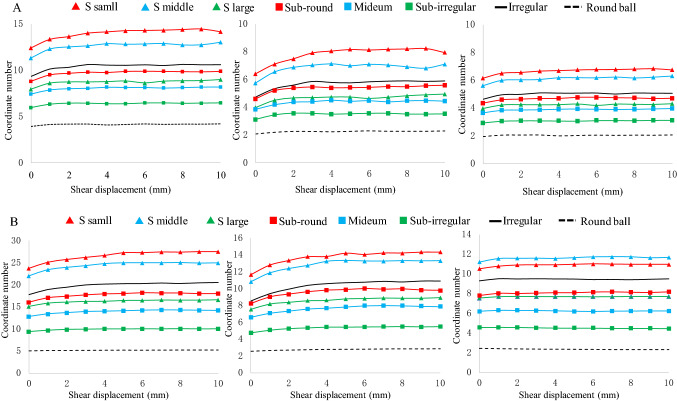
Particle coordinate number performance (**A**: 50 kPa normal stress; **B**: 200 kPa normal stress).

Figure 14 lists the particle coordinate numbers under 50 kPa and 200 kPa normal stresses. The coordinate number first increases and then quickly reaches a stable state. The coordinate number increases as the particle shape approaches an irregular shape. A good correlation can be found between the shear behavior and coordinate number. A higher coordinate number means that more contacts and interlocking structures are constructed, resulting in a steadier state. Thus, a larger shear strength can be obtained in more irregular sand^16,43^. Particles in the shear band have a larger coordinate number than the outside shear band particles. A higher normal stress also leads to a significant increase in the coordinate number. A higher coordination number leads to more sufficient contact between particles, thus leading to a particle skeleton system that can resist a higher external force.

**Figure 15:**
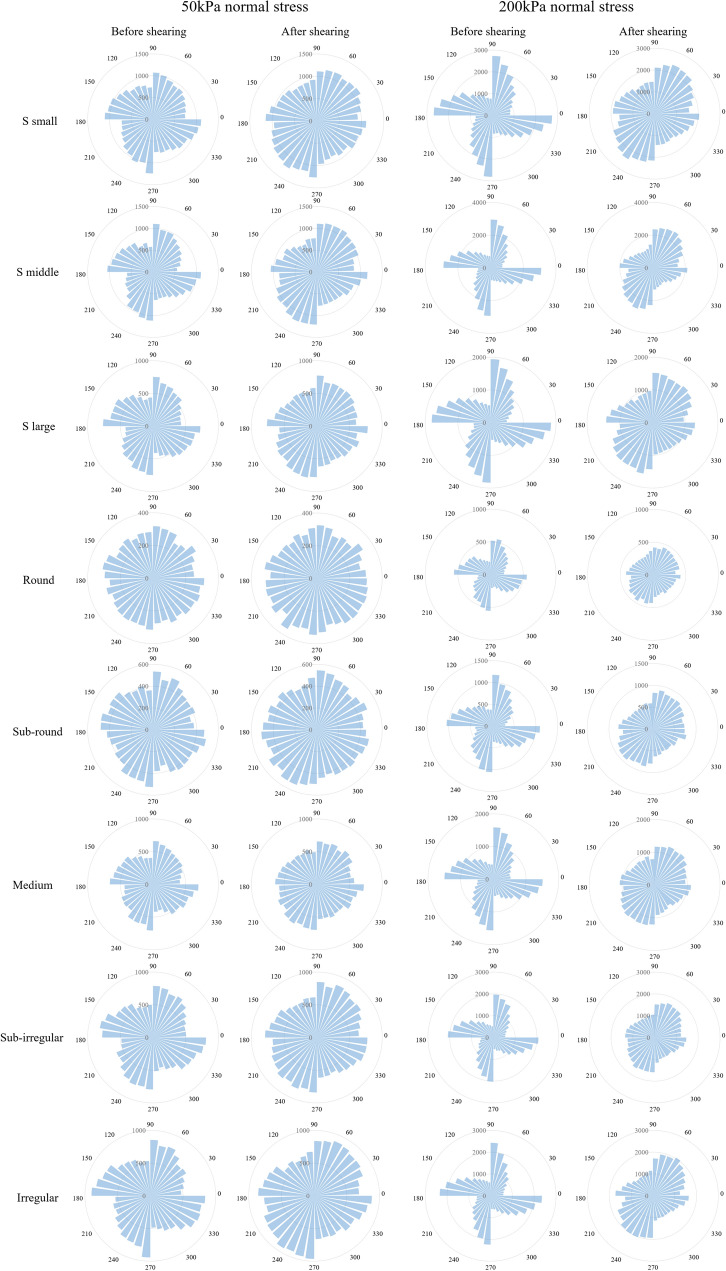
Polar distributions of contact before and after shearing.

The coordinate number shows the particle contact number performance and intergranular forces are also related to the contact orientation, which can provide information on the load transfer direction. It is known that the contact orientation of sand particles is closely related to its mechanical properties^44,45^. Under the shear test, the distribution of internal contact force depends on the contact orientation^46^. Thus, to obtain the sand particle orientation anisotropy performance during shear, the contact orientation distributions of the specimens before and after shearing under 50 kPa normal stress are plotted in Fig. 15. At lower normal stress, round particles have an approximately isotropic contact direction before shear, but the contact direction becomes anisotropic when the particle shape becomes irregular. They present a windmill form with maximum contact numbers in the 0°, 90°, 180° and 270° directions. Because the specimen is confined by the rectangular shear box, only vertical stress is applied on the top plate of the shear box. The contact forces in the perpendicular and horizontal directions are dominant. In addition, the contact numbers of irregular sand particles are much higher than those of round sand. Because there is only one contact between two individual round particles, more contacts can be generated in the clumps due to their irregular shape. When the specimen is subjected to horizontal shear, the orientation of the contact force particles in the shear box begins to change. Because particles in the lower shear box are forced to move by the left plate, they are subjected to both vertical and horizontal forces simultaneously, leading to an increase in the contact number from 30° to 70° and 210° to 240°, respectively. This is consistent with the force chain distribution in Fig. 13, where the force chain presents a diagonal distribution after shear.

Soil structure is the combination of soil composition, particle spatial arrangement and interparticle forces which results in the anisotropy^47^. The alignment direction of the particle leads to different contact forces and strengths. To quantitatively analyze the contact direction and the structural anisotropy of the corresponding material, a fabric tensor describing the particle arrangement and anisotropy is given as follows:

2 $${F}_{ij}=\frac{1}{N}\sum_{k=1}^{N}{n}_{i}^{\left(k\right)}{n}_{j}^{\left(k\right)} \quad i,j=\mathrm{1,2}$$

where *N* is the contact number and *n*_*i*_ and *n*_*j*_ are the components of the unit branch vector in the *i* and *j* two-dimensional directions, respectively.

In the two-dimensional plane, the fabric tensor can be expressed as:

3 $${\overline{F} }_{ij}=\left[\begin{array}{ll}{\overline{F} }_{11}& {\overline{F} }_{13}\\ {\overline{F} }_{31}&{\overline{F} }_{33}\end{array}\right]=\left[\begin{array}{ll}\frac{1}{N}\sum_{k=1}^{N}{sin}^{2}({\theta }^{(k)})& \frac{1}{N}\sum_{k=1}^{N}cos({\theta }^{(k)})sin({\theta }^{(k)})\\ \frac{1}{N}\sum_{k=1}^{N}cos({\theta }^{(k)})sin({\theta }^{(k)})& \frac{1}{N}\sum_{k=1}^{N}{cos}^{2}({\theta }^{(k)})\end{array}\right]$$

where $cos\left({\theta }^{(k)}\right)$ and $sin\left({\theta }^{(k)}\right)$ are *k* particle contact normal vectors in the *i* and *j* directions, respectively.

${\overline{F} }_{ij}$ is a symmetric second-order tensor. According to the material plane stress state analysis method, the major fabric ${\overline{F} }_{1}$ and minor fabric ${\overline{F} }_{3}$ of ${\overline{F} }_{ij}$ can be expressed as:

4 $${\overline{F} }_{1}, {\overline{F} }_{3}=\frac{1}{2}({\overline{F} }_{11}+{\overline{F} }_{33})\pm {\left[{\frac{1}{4}({\overline{F} }_{11}-{\overline{F} }_{33})}^{2}+{{\overline{F} }_{13}}^{2}\right]}^\frac{1}{2}$$

Then, combining Eqs. (3) and (4), the following can be obtained:

5 $${\overline{F} }_{1} , {\overline{F} }_{3}=\frac{1}{2}\pm {\left[{\frac{1}{4}({\overline{F} }_{11}-{\overline{F} }_{33})}^{2}+{{\overline{F} }_{13}}^{2}\right]}^\frac{1}{2}=\frac{1}{2}\pm \frac{{a}_{1}}{2}$$

where $${a}_{1}=\frac{1}{N}{\left\{{\left[\sum_{k=1}^{N}\left({cos}^{2}\left({\theta }^{(k)}\right)-{sin}^{2}({\theta }^{(k)})\right)\right]}^{2}+{\left[\sum_{k=1}^{N}sin\left(2{\theta }^{(k)}\right)\right]}^{2}\right\}}^\frac{1}{2}$$.

**Figure 16:**
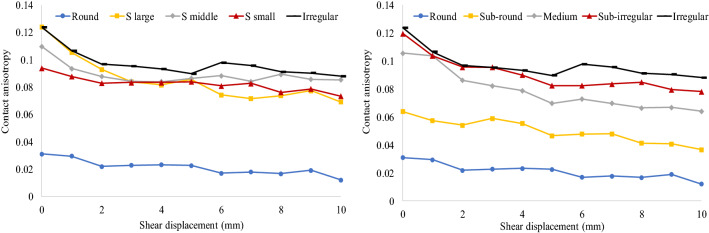
Contact anisotropy of each simulation at 50 kPa normal stress.

It is an anisotropy amplitude parameter that can be calculated to evaluate contact anisotropy. Figure 16 shows the contact anisotropy of each simulation at a 50 kPa normal stress. When particles are subjected to shear, the contact anisotropy value has a stable downward trend, highlighting the evolution of contact anisotropy. It can be seen from the left figure that round particles have the smallest contact anisotropy value, while irregular clumps have the maximum value. For the single shape particles, they all present anisotropic behavior. Contact anisotropy may not be directly linked to the S value as they (S value small, middle and large) all have similar values of contact anisotropy. On the other hand, the contact anisotropy value of the mixture containing round particles and irregular clumps in the right figure also shows a stable downward trend. However, with increasing the content of round particles, the contact anisotropy value decreases.

## Conclusion

A discrete element simulation on direct shear performances of different particle shapes is presented. The sand particle shape is first captured by a microscope and then modeled by the particle flow code. Two methods are adapted to simulate different approaching methods for round to irregular sand. To analyze the micro behavior of sand under shear, we define the shear band proportion. This allows us to analyze the particle shear performance within or without a shear band. The shear stress–displacement relationship, particle displacement, shear band proportion, particle rotation, particle contact force chain, contact coordinate, and anisotropy are analyzed to evaluate the shear performance of sand with different shapes.

At lower normal stress, irregular sand shows higher shear strength than round particles. When the particle shape approaches a round figure, its shape has less influence on the shear strength at higher normal stress. The visualization of the shear band indicates that the shear band in the shear box has a fusiform shape with a thick shear band in the middle. The quantitative analysis of the shear band area proportion shows that the shear band proportion is not a constant value for each specimen; it changes according to the shear displacement. The irregular shape of sand leads to an increase in the shear band proportion. The mixture of irregular and round sand significantly increases the shear proportion, especially at higher normal stresses. Regardless of shear displacement and normal stress, particles within the shear band have a larger average rotation angle than those outside the shear band. Irregular particle shape reduces particle rotation. The coordinate number increases as the particle shape forms into an irregular shape. A good correlation can be found between the shear behavior and coordinate number. A higher coordinate number means that more contacts and interlocking structures are constructed, resulting in a steadier state. Round particles have an approximately isotropic contact direction, but it changes to anisotropy when the particle shape gradually changes.
